# Binding of an Indenoisoquinoline to the Topoisomerase-DNA Complex Induces Reduction of Linker Mobility and Strengthening of Protein-DNA Interaction

**DOI:** 10.1371/journal.pone.0051354

**Published:** 2012-12-06

**Authors:** Giordano Mancini, Ilda D’Annessa, Andrea Coletta, Giovanni Chillemi, Yves Pommier, Mark Cushman, Alessandro Desideri

**Affiliations:** 1 CASPUR Inter-University Consortium for the Application of Super-Computing for Universities and Research, Rome, Italy; 2 Department of Biology and Centro di Bioinformatica e Biostatistica, University of Rome Tor Vergata, Via Della Ricerca Scientifica, Rome, Italy; 3 Laboratory of Molecular Pharmacology, National Cancer Institute, Bethesda, Maryland, United States of America; 4 Department of Medicinal Chemistry and Molecular Pharmacology, College of Pharmacy, and The Purdue Center for Cancer Research, Purdue University, West Lafayette, Indiana, United States of America; IISER-TVM, India

## Abstract

Long-duration comparative molecular dynamics simulations of the DNA-topoisomerase binary and DNA-topoisomerase-indenoisoquinoline ternary complexes have been carried out. The analyses demonstrated the role of the drug in conformationally stabilizing the protein-DNA interaction. In detail, the protein lips, clamping the DNA substrate, interact more tightly in the ternary complex than in the binary one. The drug also reduces the conformational space sampled by the protein linker domain through an increased interaction with the helix bundle proximal to the active site. A similar alteration of linker domain dynamics has been observed in a precedent work for topotecan but the molecular mechanisms were different if compared to those described in this work. Finally, the indenoisoquinoline keeps Lys532 far from the DNA, making it unable to participate in the religation reaction, indicating that both short- and long-range interactions contribute to the drug poisoning effect.

## Introduction

**Figure 1:**
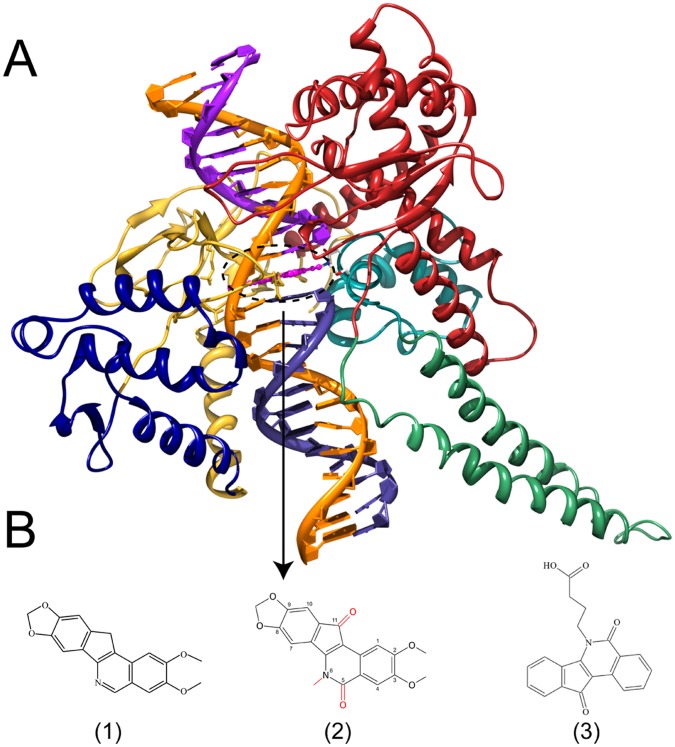
Structure of the ternary complex. (**A**) three-dimensional structure of the hTopIB-DNA-IQN2 ternary complex. Topoisomerase core subdomains I, II and III are represented in blue, yellow and red, respectively, while the linker and C-terminal domains in green and cyan, respectively. DNA strands are colored in orange (uncleaved strand), purple (cleaved upstream) and dark purple (cleaved downstream). IQN2 is shown in magenta using ball and stick representation. Chemical structures of AI-III-52 (**1**), NSC 314622 (IQN2) (**2**) and MJ-238 (**3**).

Human topoisomerase IB (hTopIB) is a nuclear enzyme whose role is to control the topological state of DNA. It catalyzes the relaxation of supercoiled substrates in order to permit fundamental cellular processes, such as transcription and replication, [1], [2]. The enzyme is composed of 765 residues divided into an N-terminal domain (residues 1–214), a core domain (residues 215–635) further subdivided into subdomain I (residues 215–232 and 320–433), subdomain II (residues 233–319) and subdomain III (residues 434–635), a linker domain (residues 636–712) and a C-terminal domain (residues 713–765) [3]–[5]. The protein has a globular shape, with subdomains I and II forming the CAP domain and subdomain III and C-terminal domain forming the CAT domain (Figure 1A) [4]. The linker domain is formed by two long α-helices that extrude outside from the globular shape of the protein (Figure 1A).

hTopIB resolves DNA supercoils by a mechanism defined as controlled rotation, which is composed by 5 steps: 1) the protein wraps around the substrate forming a non-covalent hTopIB-DNA complex, which is stabilized by intra- and inter-molecular hydrogen bonds and electrostatic interactions; 2) the catalytic Tyr723, assisted by the other four residues composing the catalytic pentad (Arg488, Lys532, Arg590 and His632), carries out a nucleophilic attack on one strand of the dsDNA forming a covalent protein-DNA complex at the 3′ end, termed the cleavage complex; 3) the broken strand rotates around the unbroken strand, controlled by the interaction with the linker domain, relaxing the supercoil; 4) the broken strand is religated; 5) the protein is released [5].

hTopIB has a relevant clinical role, being highly active in cells with a high level of duplication, i.e. cancer cells, and being the unique target of the camptothecins (CPTs), a class of natural compounds with anticancer activity [6]–[8]. CPT, the parental compound of the class, acts by stabilizing the cleavage complex and preventing the religation step. The binding of CPTs to the cleavage complex is per se reversible, but the compound becomes lethal due to the collision of the stalled enzyme-DNA complex with the replication fork [9]–[11]. CPT and its derivatives, such as topotecan (TPT), bind the binary complex intercalating between the bases of the DNA substrate at the cleavage site. The ternary complex is further stabilized by interactions of the drug with the protein, as shown by the X-ray structure of the protein-DNA-drug ternary complexes [12], [13]. Molecular dynamics (MD) simulation of the hTopIB-DNA-TPT ternary complex has highlighted the elements important for the TPT stabilization and has revealed an unusual rigidity of the linker domain when compared to the binary complex [14]. Recently new hTopIB inhibitors have been developed and among them the indenoisoquinoline (IQN) derivatives appear to be the most promising ones [14]–[16]. They stabilize the cleavage complex through a similar intercalation binding mechanism displayed by CPTs, but they seem more promising for clinical use due to their chemical stability, while CPT undergoes inactivation by lactone ring hydrolysis at physiological pH. The indenoisoquinolines also display a more persistent stabilization of the cleavage complex than CPTs and that they do not represent a substrate for the ABCG2 efflux pump [16]–[19].

The first IQN synthesized and tested against hTopIB was NSC 314622 [20], followed by the synthesis of several derivatives, two of them, NSC 725776 and NSC 724998, being now in phase I clinical trials [18]. Up to now the crystal structures of the ternary complexes with IQN derivatives have been solved only in presence of AI-III-52 and MJ-238 (structures 1 and 3 in Figure 1B) [12], [21], [22]. In the case of the MJ-238 compound, the drug is intercalated in the DNA substrate with the substituent on the nitrogen atom facing the DNA major groove, due to the steric hindrance of the substituent [12]. In the case of the compound AI-III-52 the orientation of the drug in the binding pocket is flipped 180° with respect to first one, with the nitrogen atom facing the DNA minor groove [21], [22].

No 3D structure is available for the ternary complex with NSC 314622, which represents the lead compound of IQNs already in clinical trial (NSC 725776 and NSC 724998). NSC 314622 displays a moderate anti-topoisomerase activity [23] and differs from AI-III-52 in the presence of two oxygen substituent in position 5 and 11 and a methyl substituent in position 6 (structure 2 in Figure 1B). The evaluation of its mode of binding can provide useful information on its mechanism of stabilization of the cleavage complex. To this aim the compound NSC 314622, termed IQN2, has been modeled taking the ternary complex in presence of AI-III-52 as a template and 75 ns comparative molecular dynamics simulations of the hTopIB-DNA binary and hTopIB-DNA-IQN2 ternary complexes have been carried out. The results show that the drug conformationally stabilizes the protein-DNA complex reducing the fluctuation of the lips and decreases the conformational space sampled by the linker domain due to the increased compactness of the helix bundle proximal to the active site.

## Materials and Methods

The initial configuration of hTopIB, in covalent complex with a 22 base pair linear double helix DNA substrate, has been modeled from the crystallographic structures of the binary and ternary complexes (PDB 1K4S and 1TL8, respectively) [13], [22]. For the binary complex the starting positions for residues 201–631 and 708–765 have been obtained from the 1K4S crystal structure and those for residues 632–707 from the 1TL8 crystal structure (since the linker domain is not resolved in the former), following a mass-weighted fit of backbone atoms on 1K4S that gives rise to an RMSD between the two structures of 0.5 Å after the fit. As far as the ternary complex is concerned, both the 1SC7 and 1TL8 3D structures may be considered as a template (12,22), the latter one being selected since the dimension of the AI-III-52, present in the 1TL8 structure, and the NSC 314622 (IQN2) compounds are very similar and both lack a butyric substituent on the nitrogen atom in position 6 (Figure 1). The same 22 base pair DNA sequence of the ternary complex was used in both systems (nucleotides in relevant positions in the binary system were mutated using the rotamer module presents in the Chimera package [24]). The systems have been modeled using the AMBER03 all-atom force field [25] implemented by Sorin and Pande [26] in the GROMACS MD package version 4.0.7 [27]. The protein has been immersed in a rhombic dodecahedron box with a minimum distance of 14 Å from the box edges. The box was then filled with water molecules described by means of the TIP3P rigid potential [28] and Na^+^ counter-ions have been added to neutralize DNA-enzyme complex total charge using the genion tool of the GROMACS package, which randomly substitutes water molecules with ions at the most favorable electrostatic potential positions. The resulting systems are composed of 9456 protein atoms, 1400 DNA atoms, 58919 water molecules, 20 Na^+^ ions and one IQN2 molecule in the ternary complex, for a total of 187633 and 187591 atoms in the ternary and binary systems, respectively. Electrostatic interactions have been taken into account by means of the Particle Mesh Ewald method (PME) using a cutoff of 1.2 nm for the real space and Van der Waals interactions [29], [30]. The LINCS algorithm was used to constrain bond lengths and angles [31]. Relaxation of solvent molecules and Na^+^ ions was initially performed keeping solute atoms restrained to their initial positions with a force constant of 1000 kJ/(mol • nm), for 3500 ps. The two systems have then been simulated for 75 ns with a time step of 2.0 fs and the neighbor list was updated every 10 steps. Temperature was kept constant at 300 K using the velocity rescale method with a coupling constant of 0.1 ps during sampling, while pressure was kept constant at 1 bar using the Parrinello-Rahman barostat with a coupling constant of 1.0 ps during sampling [32], [33].

Root mean square deviations (RMSD) were calculated using the following formula (after a mass-weighted least square fitting to a reference structure):
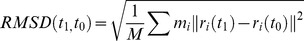
where *M* is the sum of atomic masses, *m_i_* is the mass of atom *i* and *t = 0* refers to the selected reference structure. The per-residue root mean square fluctuations (RMSF) were computed using the following equation:



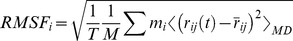
where the averages have been calculated over the equilibrated MD trajectories.

Principal components analysis (PCA) was carried out on the 3N×3N cartesian displacement matrix whose elements are calculated as:
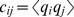
where N is the number of Cα protein or C5′ DNA atoms of the two systems and *q_i_* is the (mass-weighted) displacement of the *i-th* Cα protein or C5′ DNA atoms from the reference value (after removal of rotational and translational degrees of freedom). The first few eigenvectors of the diagonalized covariance matrix usually account for a major fraction of the total variance and projection of atomic trajectories over the corresponding eigenvectors represents large collective atomic motions. The cosine content (*c_i_*) of a principal component *p_i_* is a good indicator for good simulation sampling [33]. It ranges between 0 and 1 and is calculate in the following way:



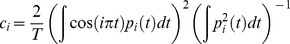
where t and T are instantaneous and total simulation time, respectively. High values of *c_i_* (close to 1) are indicative of random diffusion motion and therefore insufficient sampling. Cosine contents along these eigenvectors have maximum values of 0.11 and 0.27 for the binary and ternary complex, respectively, (with the exception of principal component 3 in the binary trajectory that has a cosine content of 0.55; however this eigenvector accounts only for about 3% of total variance) indicating a satisfactory convergence of simulations along these principal components.

To compare the two simulations, sampled structures were clustered considering only the backbone atoms using the GROMOS method: after the construction of the M×M RMSD matrix (where M is the number of sampled structures). The structure with the largest number of neighbors (i.e. configurations within the cutoff range) is taken as the centroid of the first cluster and it is eliminated by the pool with all its neighbors; the process is repeated until all structures have been assigned to a cluster. A total of 7000 frames from each simulation were selected with constant pace of 10 ps to ensure absence of correlation between neighbor frames and then used in the cluster analysis.

All analyses have been carried out with standard tools present in the GROMACS MD package v. 4.0.7 [27] or in-house written codes, except for secondary structure assignment, which was performed by means of the DSSP program [34]. Graphs have been obtained with the Grace program (Grace. http://plasma-gate.weizmann.ac.il/Grace/) and images have been created using the VMD [35] and Chimera packages [24].

## Results

### Protein and Linker Mobility

**Figure 2:**
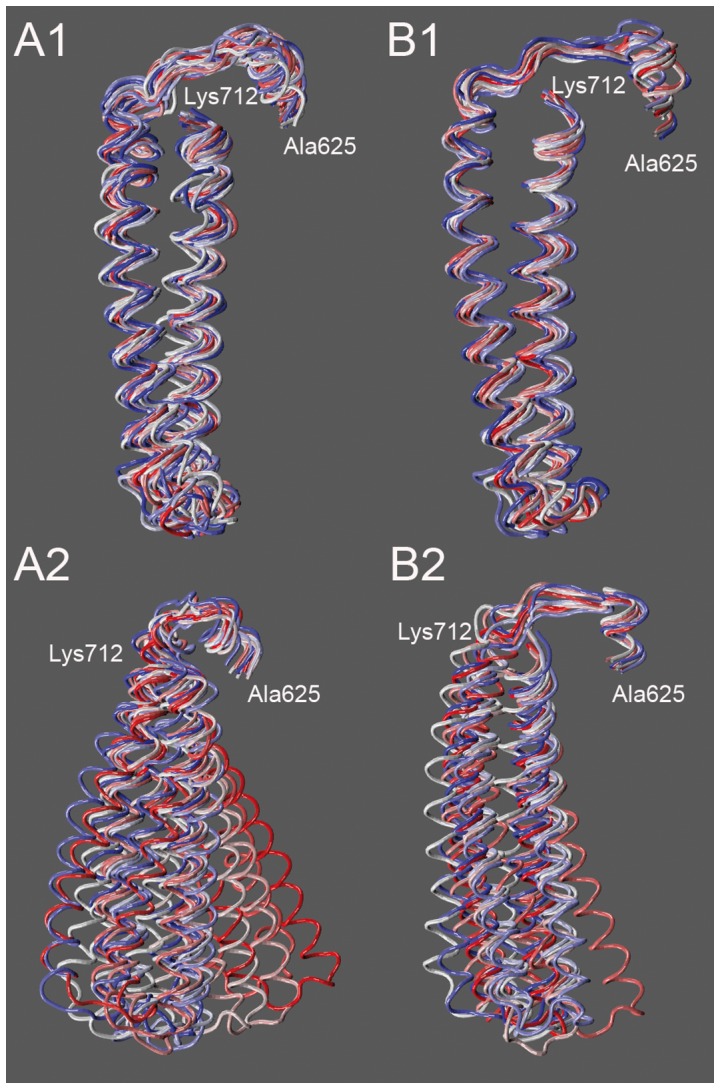
Cluster analysis. Overlap of the linker representative clusters in the binary (**A1 and A2**) and ternary (**B1 and B2**) complexes. The linker is represented as a coil and the color scale is red-white-blue from the most to the less populated cluster. On the top (A1 and B1) the representative structures obtained upon fitting on the linker itself are reported, while on the bottom (A2 an B2) the representative structures obtained upon fitting on the core and C-terminal domains of the protein.

RMSD calculated on the Cα atoms of hTopIB in the binary and ternary complexes shows that in both cases the protein displays a highly oscillating behaviour up to a value of 4 and 3 Å, respectively (Supporting Figure S1, dashed lines). On the other hand, once the contribution of the linker domain is excluded from the calculation the RMSD reaches a plateau slightly larger in the binary than in the ternary complex (Supporting Figure S1, full lines). These data indicate that the linker is the most mobile domain of the protein and demonstrates that the presence of the drug has a remarkable stabilizing effect. Clustering of the protein structures extracted from the simulations, using a cut off of 2.5 Å, gives rise to 10 and 3 clusters for the binary and ternary complexes respectively, confirming that the protein in presence of IQN2 samples a reduced conformational space. The first three clusters of the binary complex account for 85% of the total structures, while in the ternary complex 99% of the conformations are represented by the first cluster, highlighting the extreme rigidity of the protein stabilized by the drug. Clustering of the linker domain using a cut off of 1.6 Å and fitting the linker on itself highlights the different conformational space sampled by this domain in the two systems, giving rise to 33 clusters in the binary complex and 13 in the ternary one (Figure 2A1 and 2B1). In this case 90% of the total conformations reside in the first 5 and 2 clusters for the binary and ternary simulations, respectively. Clustering of the relative orientations of the linker respect to the core domain, using the same cut off, gives rise to 15 clusters in the binary complex and 11 in the ternary one (Fig. 2A2 and 2B2). As a result f this clustering 90% of the total conformations belong to the first 3 clusters for the binary complex and to the first cluster for the ternary one.

Principal component analysis carried out on the Cα atoms shows that 60% of total motion is represented by the first 4 eigenvectors in the binary complex indicating (Supporting Figure S2), indicating that 60% of the total motion is spread toward four different directions, while in the presence of IQN2 the same percentage of motion is described by the first eigenvector, confirming that the motion of the protein is core confined in the presence of the drug (Supporting Figure S2). This can be well appreciated by the plot of the projection of the Cα atoms trajectories on the plane formed by the first and second eigenvectors (Supporting Figure S3A) and by the first and third eigenvectors (Supporting Figure S3B), both showing that the protein in the ternary complex samples a low conformational space being the motion confined in a single basin, while a much larger space is sampled by the protein in the binary complex (Supporting Figure S3). In line, the amplitude of motion along the first eigenvector is 193 Å and 117 Å for the binary and ternary complex, respectively (Supporting Figure S3).

**Figure 3:**
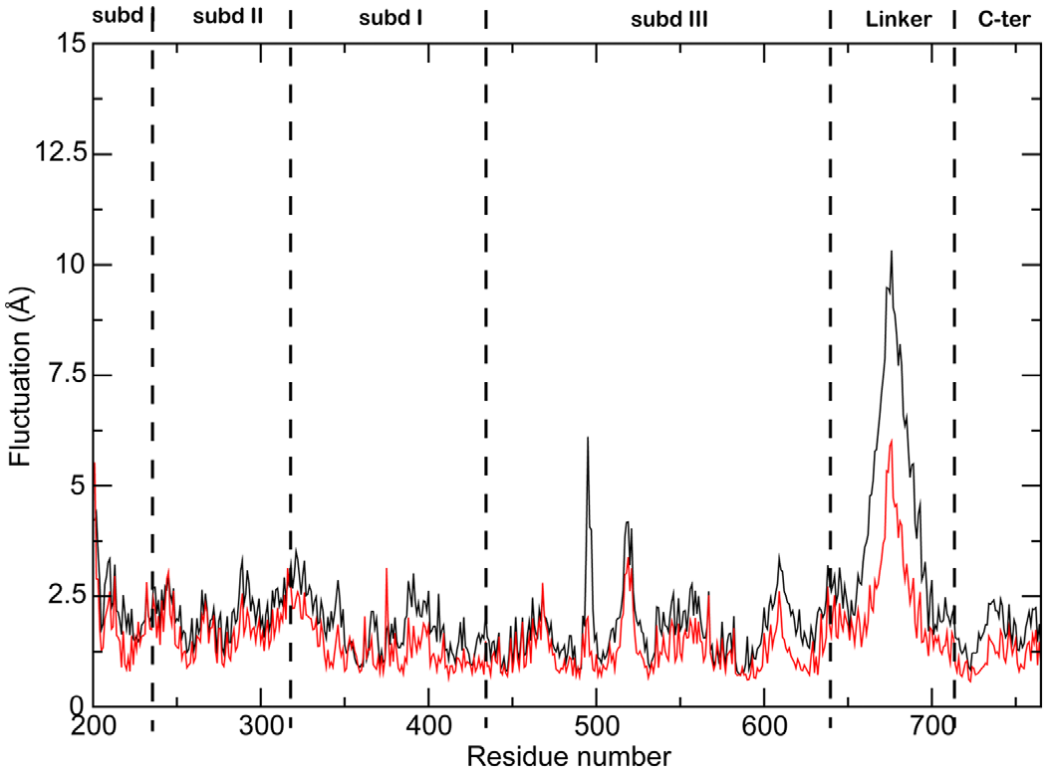
Root Mean Square Fluctuation. Per-residue root mean square fluctuation (RMSF) of binary (full black line) and ternary (red dot-dashed line) complex trajectories calculated along the entire sampling interval (75 ns).

**Figure 4:**
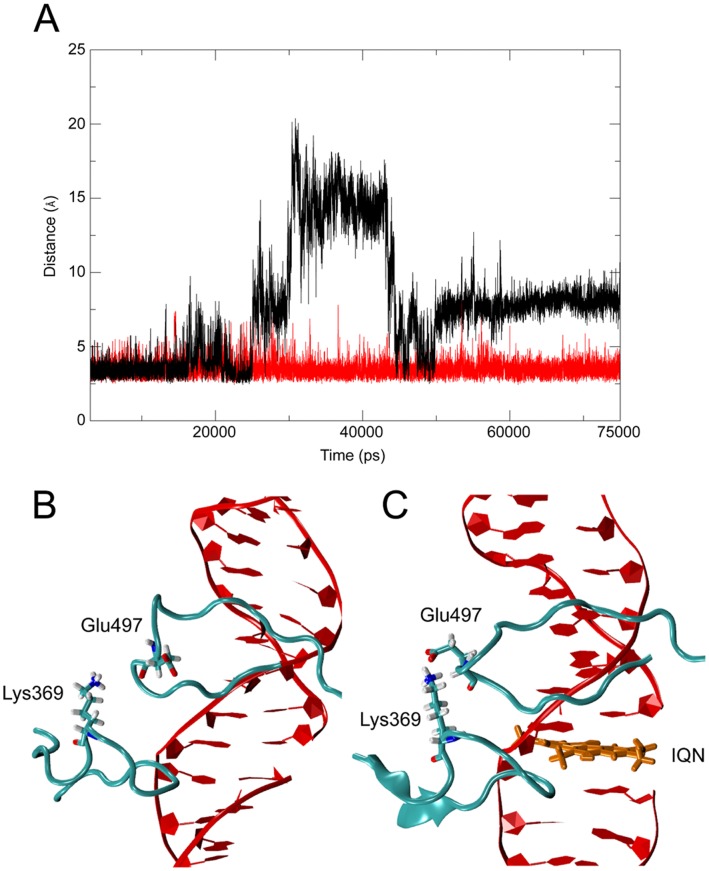
Interaction between the lips. (**A**) atomic distance between the Nζ atom of Lys369 and the center of mass of OΕ1 and OE2 of Glu497 as a function of time in the binary (black line) and ternary (red line) complexes. Representative snapshots extracted from the simulations of the binary (**B**) and ternary (**C**) complexes showing the relative positions of Lips 1 and 2 and of residues Lys369 and Glu497.

A reduced linker flexibility in the ternary complex is also confirmed by the plot of the per-residue RMSF calculation (Figure 3) of the binary and ternary complex. The plot shows that also residues 587–630, corresponding to helices 16 and 17 in the C-terminal portion of subdomain III, and residues 717–765, corresponding to helices 20–22 in the C-terminal domain, are less flexible in the ternary complex. Another large difference is detected at the level of Glu497, a residue belonging to the Lip2 that, from the X-ray diffraction study of the binary complex, is known to clamp the DNA substrate interacting via a salt bridge with Lys369 belonging to Lip1 [3], [4]. Analysis of the MD trajectories indicates that this interaction is present for 100% of total simulation time in the ternary complex, but for only 36% of simulation time in the binary one, where several conformations are reached. A plot of the distance between the carboxylic group of Glu497 and the amino group of Lys369 as a function of time indicates that this distance is highly fluctuating, since the interaction is partially lost after 18 ns and then occurs again at around 20 ns for 2 ns, and then is lost again, the two groups reaching a distance up to 20 Å (Figure 4A). Two representative conformations extracted from the binary and ternary complex are shown in Figure 4B and C to better appreciate the difference between the two systems, where the two residues are at a distance of 20 Å and 2.4 Å, respectively. The loss of the inter-lips interaction explains the high mobility of residue 497 in the absence of IQN2 and for the first time provides a picture of the events that must continuously occur during the cleavage and religation steps carried out by the enzyme [5]. It is interesting that in the same temporal window such a process never occurs in the ternary complex, where a constant average distance of 5 Å is observed during all the trajectory (Figure 4C), indicating that the presence of the drug increases the stability of the clamp around the DNA.

### IQN2 Environment and Compactness of the Helix Bundle Proximal to the Active Site

**Figure 5:**
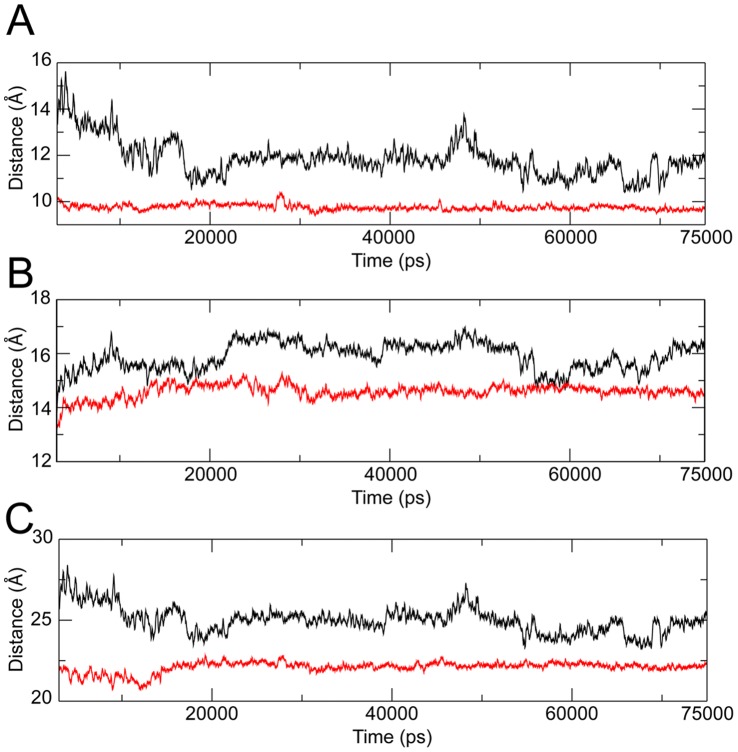
Analysis of the helix bundle. Atomic distance between the centre of masses of helices 17–19 (**A**), 19–20 (**B**) and 19–21 (*C*). The black and red lines represent the distances calculated in the binary and ternary complexes, respectively.

As shown by the plot of the RMSF, the presence of IQN2 affects not only the mobility of the linker domain, but also of helices 16–17 in core subdomain III and 20–22 in the C-terminal domain (Supporting Figure S4). The interaction between these helices has been analyzed in detail by plotting the distance between the centres of mass of the following couples of helices: 17–21, 19–20 and 19–21 (Figure 5). In the case of helix 19 only the C-terminal region, corresponding to residues 699 to 726, that directly interfaces helices 17 and 21 has been considered for the calculation (Supporting Figure S4). In all the cases the distance is larger and more fluctuating in the binary complex than in the ternary one, indicating that the presence of the drug makes the helix bundle more compact (Figure 5). The bundle is proximal to the active site and thus to the drug binding site (Supporting Figure S4), so it is directly influenced by the presence of the drug itself. To detect at molecular level the effect of the drug on the mobility of the linker and of the helices bundle, we have investigated the interactions occurring between the protein-DNA and protein-DNA-drug complexes, focusing our attention on the Asn722 and Arg364, involved in the drug binding [13], [22].

**Figure 6:**
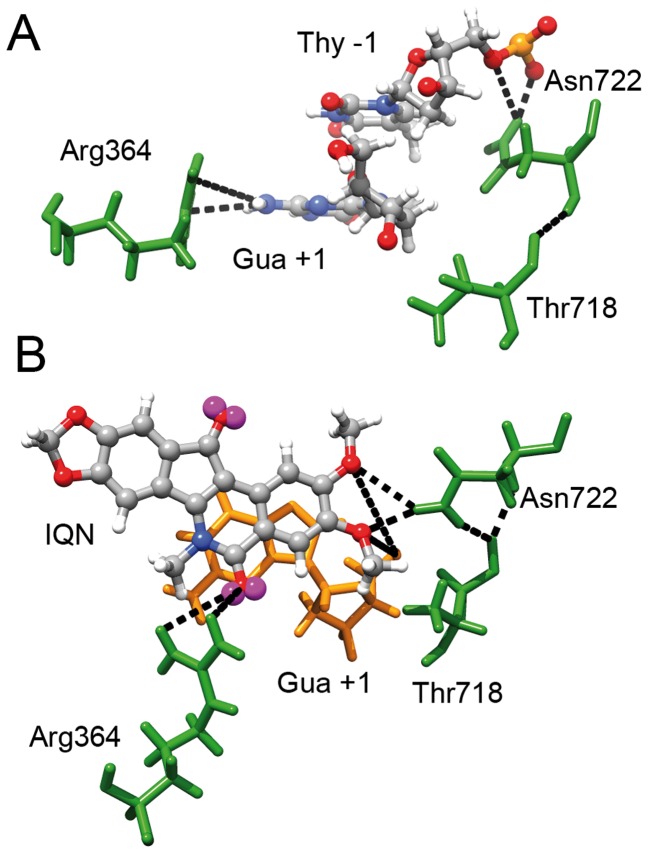
Active site and IQN interactions. Snapshots of the cleavage site in the binary and ternary complexes. Amino acids are represented in green while nucleotides are either yellow or represent in ball and stick with different colors for various atom types. Black dotted lines indicate hydrogen bonds between the drug, DNA or hTopIB residues described in the text or in Tables. (**A**) snapshot of the cleavage site in the binary complex. (**B**) snapshot of the drug binding pocket in the ternary complex.

In the crystal structure of the binary complex Asn722, located on helix 20, binds Thr718 via a backbone-backbone interaction while Arg364, located on Lip1, establishes a hydrogen bond with Gua +1 of the scissed strand [13]. In the simulation, Asn722 maintains the backbone-backbone interaction with Thr718 and establishes a direct contact with Thy -1 of the scissile strand over all the simulation time, whilst Arg364 establishes two water mediated contacts with the guanines in position +1 e +2 of the scissile strand, over all the simulation time (Table 1, Figure 6A and Supporting Figure S5A).

**Table 1:** Hydrogen bonds between hTopIB and DNA.

Donor	Acceptor		Hydrogen Bond Lifetime (%)	
**Binary**				
ARG364	G +2 s		98.5 ^wm^	
ARG364	G +1 s		90.2 ^wm^	
ASN722	T -1 s		99.6 ^wm^	
G +2 s	ARG364		91.8 ^wm^	
LYS425	C +1 u		92.6	
THR718	G +2 s		95.8	
LYS532	G +1 s		95.96 ^wm^	
**Ternary**				
SER534	G -3 u		90.7 W	
ASN574	T -6 u		92.9 ^wm^	
LYS587	C -5 u		99.9	
LYS587	T -2 s		96.6	
LYP587	T -1 s		94.4	
ARG364	IQN		96.7 ^wm^	
ASN722	IQN		96.3	
G +1 s	IQN		51.3	

Hydrogen bonds between hTopIB and DNA present in either the binary or ternary complex trajectory for at least 85% of sampling (with the exception of the last bond between the drug and the guanine residue in position +1 on the cleaved strand).

Wm ) A water molecule capable of hydrogen bonding is present at least in 50% of the analyzed frames.

W ) The indicated value refers to a water mediated hydrogen bond since a direct bond is present in less than 85 of analyzed frames.

In the crystal structure of the ternary complex in presence of the AI-III-52 compound, having the same scaffold for the here presented IQN2, the drug is stabilized by a direct interaction of the methoxy substituent in position 2 with the lateral chain of Asn722 and by a direct interaction between the nitrogen atom in position 6 and the lateral chain of Arg364 [21], [22]. In comparison to the binary complex crystal structure, Arg364 loses the interaction with Gua +1 and establishes a new interaction with Asp533, whilst Asn722 maintains the backbone-backbone interaction with Thr718 and establishes with its lateral chain, that now has new orientation induced by the drug, an additional interaction with the backbone of Thr718.

In the simulation, the interaction of the IQN2 drug with Asn722 is maintained over all the trajectory (Table 1 and Figure 6B). In the case of Arg364, its lateral chain binds the drug via a water mediated contact at the level of the of the oxygen substituent in position 5, being the N6 atom of IQN2 not available due to the presence of the methyl substituent (Table 1 and Figure 6B). The drug is further stabilized by a direct contact with DNA, at the level of the Gua +1 base of the scissile strand (Table 1 and Figure 6B). Moreover, the Asn722-Thr718 backbone-backbone and lateral chain-backbone hydrogen bonds are maintained over all the simulation time (Supporting Figure S5), avoiding the formation of the Asn722-Gua +2 and Thr718-Thy -1 hydrogen bonds observed for more than 95% of the binary complex simulation time (Table 1).

The stable interactions between Asn722 and Thr718, both located on helix 20, coupled with the constant hydrogen bond between by Asn722 and IQN2, provides an explanation for the reduced mobility of helix 20, of the bundle formed by helix 20 itself together with helices 16–17–19–21 and of the linker domain, observed in the ternary complex (Figure 3).

Another interesting difference between the binary and ternary complexes concerns the different orientation of the active site residue Lys532, that acts as a general acid in the religation step, receiving the proton from the +1 base [36]–[38]. In the binary complex Lys532 interacts with Gua +1 for the 95% of the total simulation time, while this interaction is never observed in the ternary complex (Table 1), where the Lys532-Gua +1 distance is more variable and reaches a final value of 9.8 Å, instead of the 3.2 Å constant value observed in the binary complex (Supporting Figure S6).

## Discussion

The effect of IQN2 on the structure and dynamics of the hTopIB-DNA complex has been probed by molecular dynamics simulations of the binary and ternary complexes. The results show that the presence of IQN2 affects the flexibility of the protein, reducing the conformational space visited by the linker domain, helices 16–17 in subdomain III and helices 20–22 in the C-terminal domain (Figure 3). This is due to the direct interaction between IQN2 and Asn722, located on helix 20, present for all the simulation time (Table 1 and Figure 6B). This interaction forces Asn722 to interact with Thr718 more tightly than in the binary complex, blocking the fluctuation of the helix and of the entire cluster, including the C-terminal part of helix 19, one of the two helices composing the linker domain. This domain is indeed less flexible in the ternary complex than in the binary one as evidenced by the RMSF analysis (Figure 3) and, more importantly, it samples a smaller conformational space as shown by PCA and cluster analyses (Figure 2, and Supporting Figures S2 and S3). In a previous simulation carried out in presence of topotecan (TPT), the linker was also found to sample a smaller conformational space, but in this case this was due to an increased rigidity of the loop connecting the linker with subdomain III (14) and not to an increased compactness of the helices 16–21 bundle. It is interesting to notice that the same effect, i.e. a reduction of the conformational space sampled by the linker, is achieved by the two drugs through two different mechanisms.

A direct correlation between linker mobility and TPT reactivity has been shown upon a comparison of the electron density maps of the enzyme crystals in the presence or absence of TPT [14], where the linker density is observed only in presence of the drug, confirming the fundamental role of this domain required for an efficient drug inhibition as experimentally demonstrated by Champoux and co-workers [39], [40]. The increased mobility of the linker has been shown to be the most likely explanation for the CPT resistance displayed by the mutant in which alanine at 653 is substituted with a proline [41], whilst a reduced linker mobility has been correlated to the enzyme drug hypersensitivity, observed upon multiple linker mutations [42], [43]. Recently, an increased linker structural flexibility has been suggested to explain the CPT resistance of the linker-located Glu710Gly mutation [44]. Here we show a reduction of the conformational space sampled by the linker, achieved through a direct interaction of IQN2 with helix 20, and the following tight packing of helices 16–21, including the C-terminal region of helix 19, one of the two helices forming the linker domain. The reduced protein mobility that induces a stronger inter-lips interaction (Figure 4). Actually, in this work we show for the first time that the Lys369-Glu497 salt bridge between the two lips is lost for a large time during the trajectory in the binary complex but that this never occurs in the hTopIB-DNA-IQN2 ternary complex (Figure 4), indicating the efficient conformational stabilization of the cleavage complex induced by IQN2.

**Figure 7:**
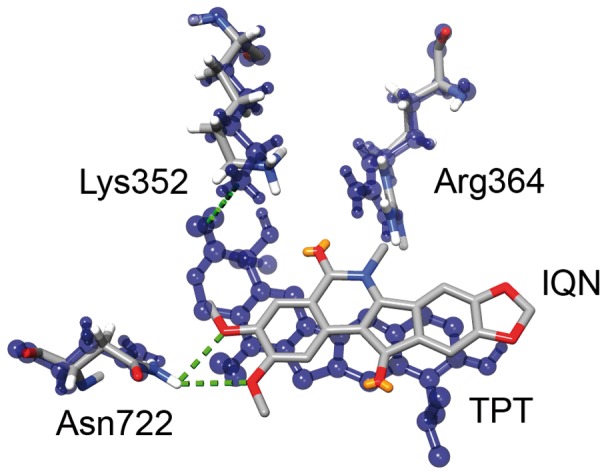
IQN and TPT binding pocket. Superposition of TPT and IQN into the DNA in the ternary complex. Thr718, Asn722 and Arg364 are shown.

An interesting observation concerns the fact that despite IQN2 and TPT display a different network of interactions with the protein, as highlighted by the superposition of the two drugs in the ternary complexes reported in Figure 7, they induce two main identical effects, even if achieved by distinct molecular mechanisms. The first one is the already described reduction of the conformational space of the linker, due to the rigidity of residues 633–644 in the case of TPT and of helices 16–21 in the case of IQN, the second one is the lengthening of the Lys532-Gua +1 distance, due to the direct interaction of residue Lys532 with TPT in one case and to the direct interaction of Gua +1 with IQN2 in the other one (Supporting Figure S6). The first event induces a reduced protein mobility and the second one impedes the religation process.

A final comment concerns the presence on IQN2 of the methoxy groups in position 2 and 3 that contribute to the religation inhibition through the reduction of protein mobility due to hydrogen bonding interaction with Asn722, (figure 6) and of the acceptor groups in position 5 or 11 that play a key role in the interaction with Arg364 (figure 6). This study indicates that these features are important requisites to conformationally stabilize the cleavage complex and to reduce linker mobility, and their presence must be considered in the design of efficient hTopIB drugs.

## Supporting Information

Figure S1 **Root Mean Square Deviation.** RMSD of Cα atoms calculated as a function of time for the full protein in the binary complex (black dashed line) and for the protein without the linker domain (black full line) and for the full protein in the ternary complex (red dashed line) and for the protein without the linker domain (red full line).
(TIF)

Figure S2 **Weight of eigenvectors.** Cumulative percentage of motion as a function of eigenvectors for the 565 Cα atoms (residues 201–765) of the protein in the binary and ternary complexes (black and red lines, respectively).
(TIF)

Figure S3 **Amplitude of the motion along the first two eigenvectors.** Projection of the motion along the planes formed by eigenvectors 1–2 (**A**) and 1–3 (**B**). The binary and ternary complexes are reported in black and red, respectively.
(TIF)

Figure S4 **Helix bundle.** Representation of the helix bundle 16–21. The protein and DNA are represented in ribbon, with the helices of the bundle reported in red.
(TIF)

Figure S5 **Time evolution of the distance between Asn722 and Thr718.** Atomic distance as a function of time between the N atom of Asn722 and the O atom of Thr718 (**A**) and between the ND atom of Asn722 and the O atom of Thr718 (**B**). In both graphs the black and red lines represent the binary and ternary complex, respectively.
(TIF)

Figure S6 **Time evolution of the distance between Lys532 and Gua +1.** Atomic distance as a function of time between the Nζ of Lys532 and the O5′ atom of Gua +1. The trajectories of the binary and ternary complexes are reported in black and red, respectively.
(TIF)
