# Supplementary figures and images for: Binding of an Indenoisoquinoline to the Topoisomerase-DNA Complex Induces Reduction of Linker Mobility and Strengthening of Protein-DNA Interaction
Source: PLoS One. 2012 Dec 6;7(12):e51354. doi: 10.1371/journal.pone.0051354 (PMC3516564; doi:10.1371/journal.pone.0051354)

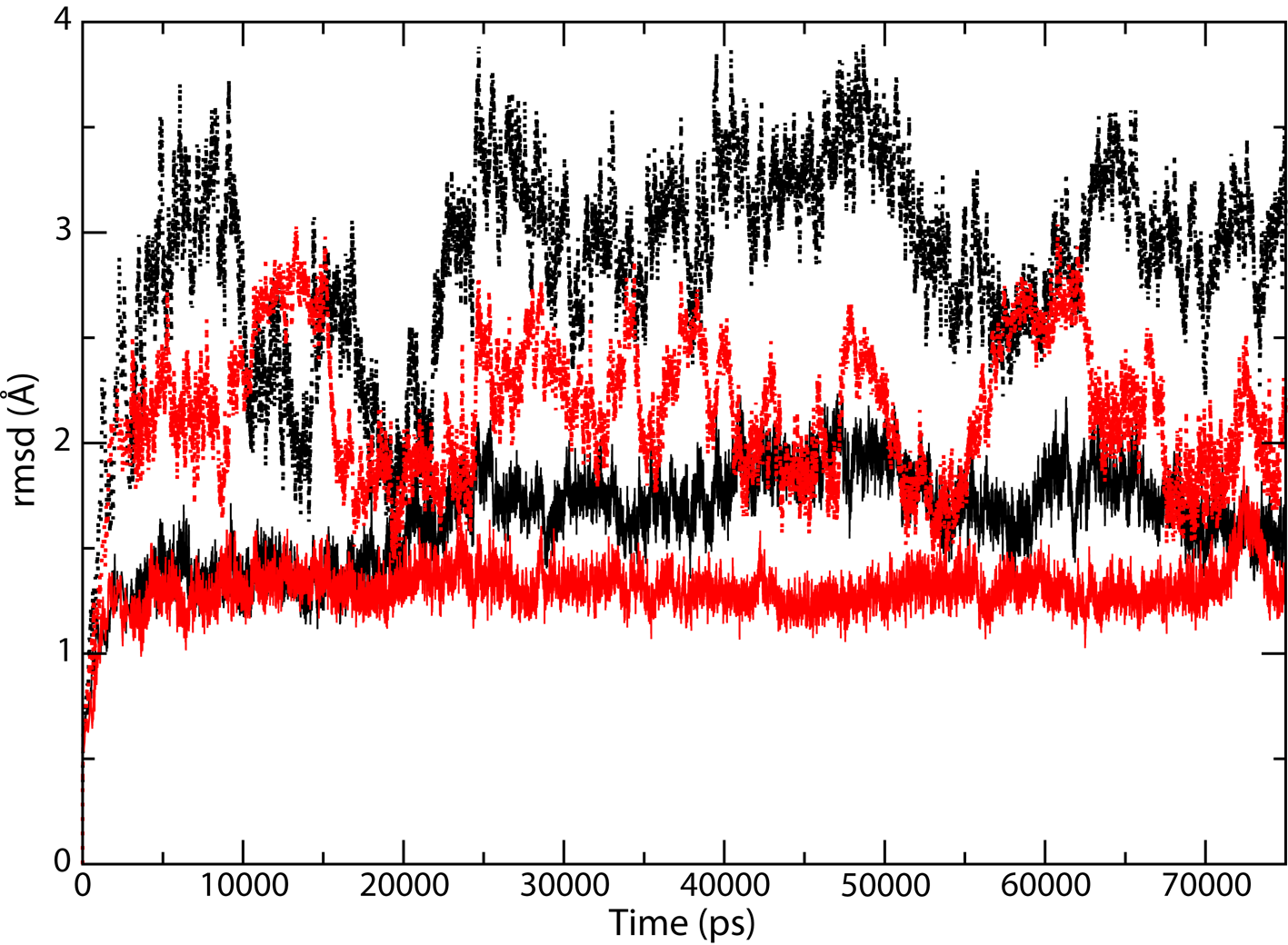

Supplement: Figure S1 — Root Mean Square Deviation. RMSD of Cα atoms calculated as a function of time for the full protein in the binary complex (black dashed line) and for the protein without the linker domain (black full line) and for the full protein in the ternary complex (red dashed line) and for the protein without the linker domain (red full line). (TIF) [file pone.0051354.s001.tif]

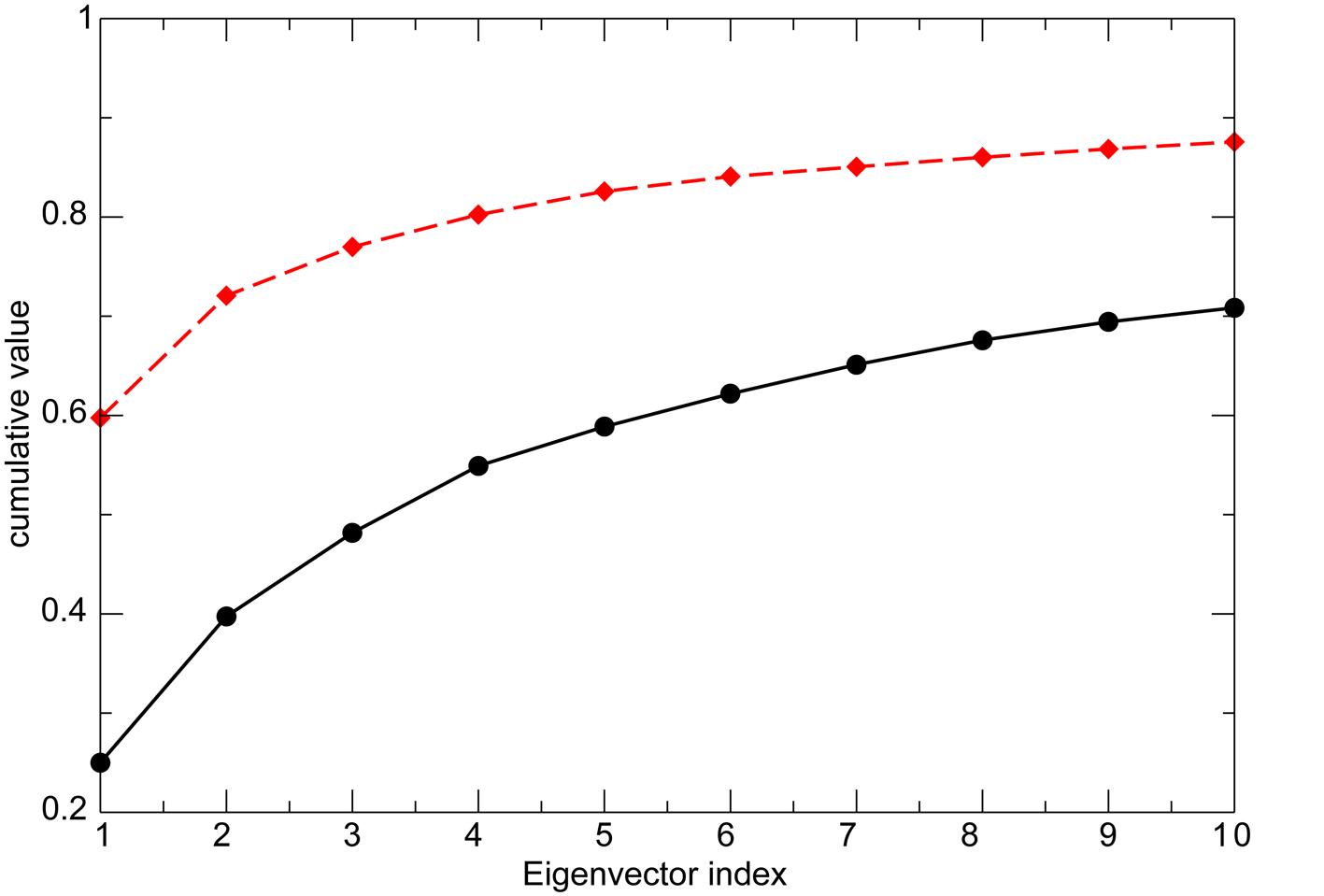

Supplement: Figure S2 — Weight of eigenvectors. Cumulative percentage of motion as a function of eigenvectors for the 565 Cα atoms (residues 201–765) of the protein in the binary and ternary complexes (black and red lines, respectively). (TIF) [file pone.0051354.s002.tif]

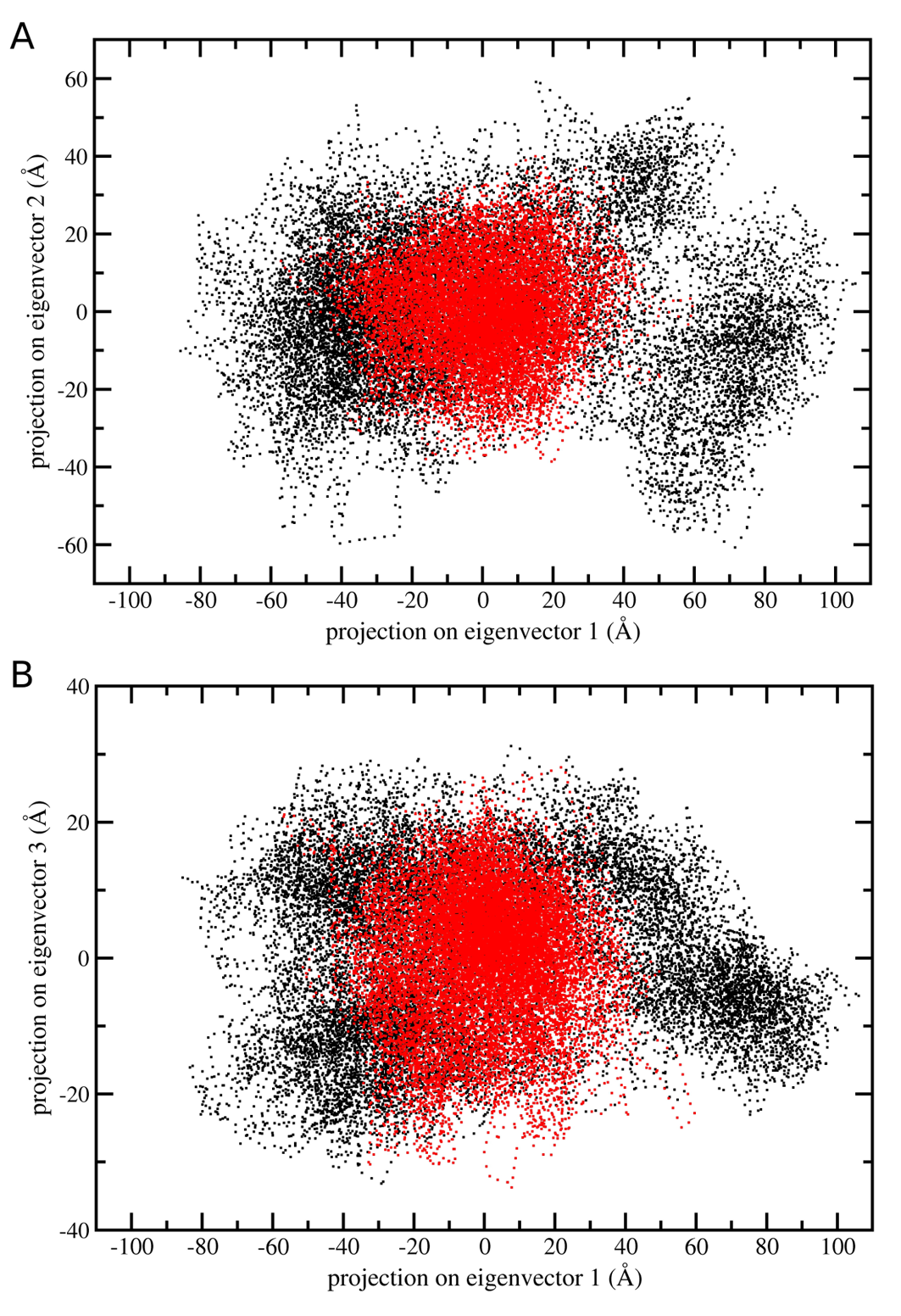

Supplement: Figure S3 — Amplitude of the motion along the first two eigenvectors. Projection of the motion along the planes formed by eigenvectors 1–2 (A) and 1–3 (B). The binary and ternary complexes are reported in black and red, respectively. (TIF) [file pone.0051354.s003.tif]

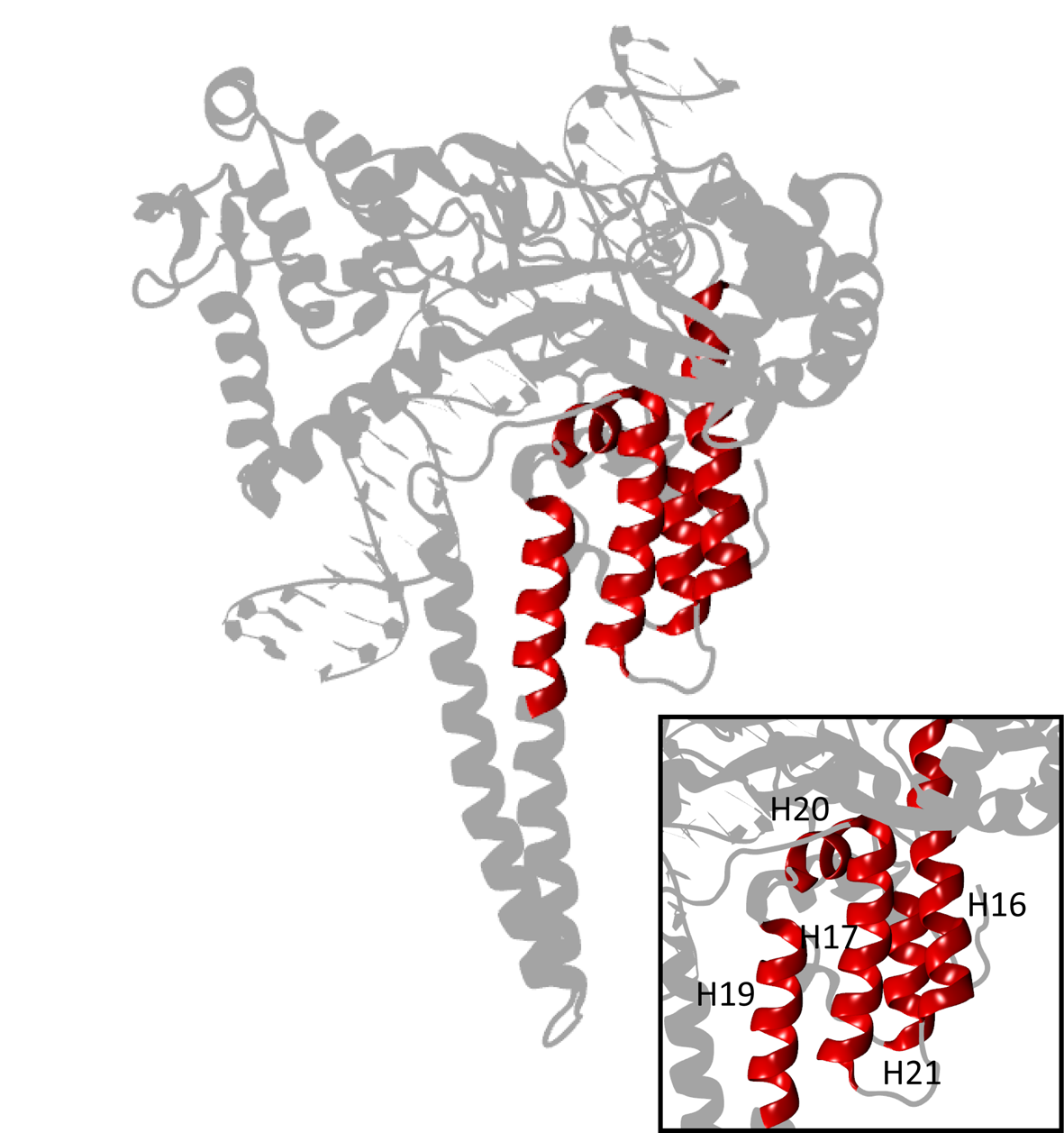

Supplement: Figure S4 — Helix bundle. Representation of the helix bundle 16–21. The protein and DNA are represented in ribbon, with the helices of the bundle reported in red. (TIF) [file pone.0051354.s004.tif]

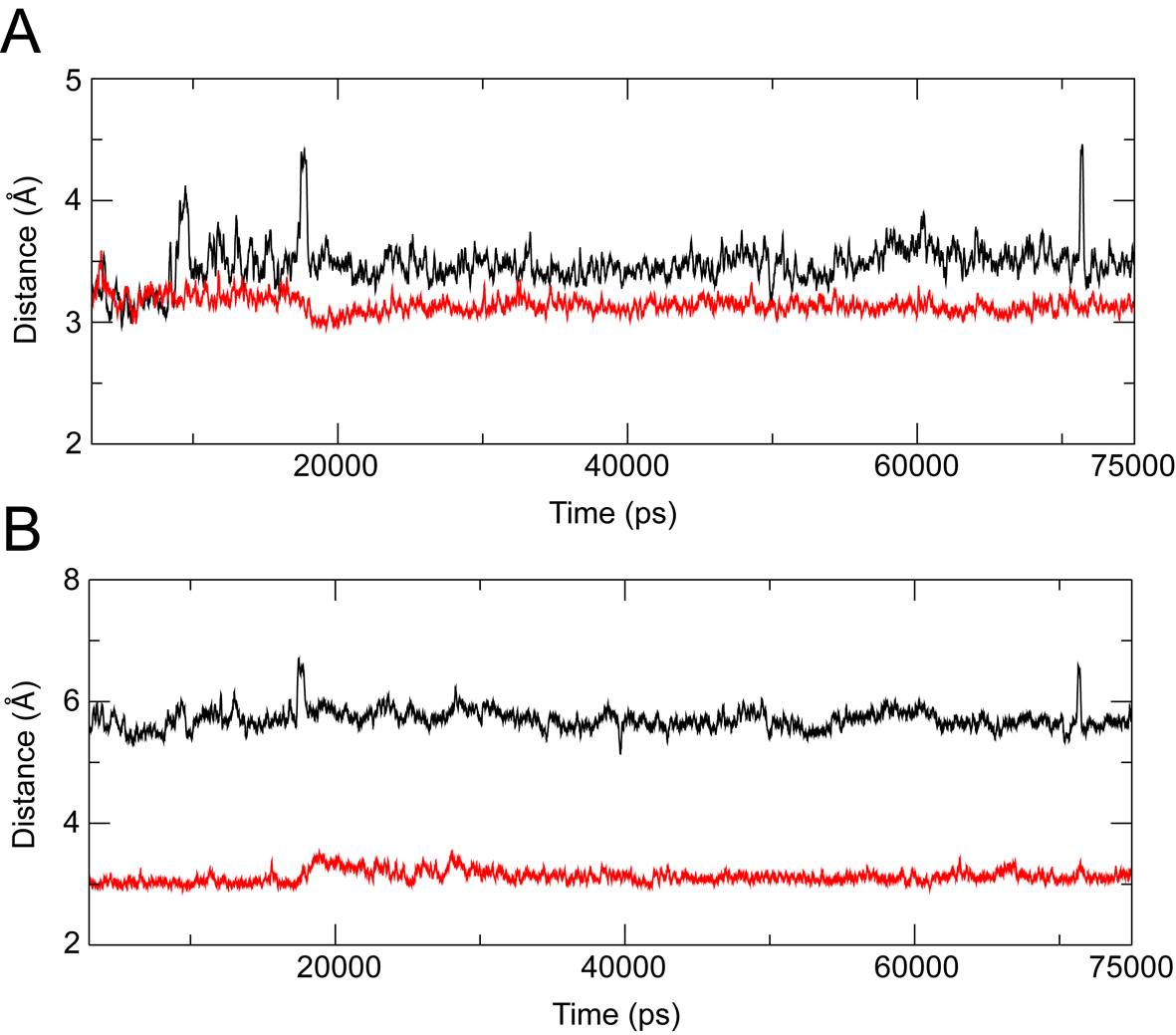

Supplement: Figure S5 — Time evolution of the distance between Asn722 and Thr718. Atomic distance as a function of time between the N atom of Asn722 and the O atom of Thr718 (A) and between the ND atom of Asn722 and the O atom of Thr718 (B). In both graphs the black and red lines represent the binary and ternary complex, respectively. (TIF) [file pone.0051354.s005.tif]

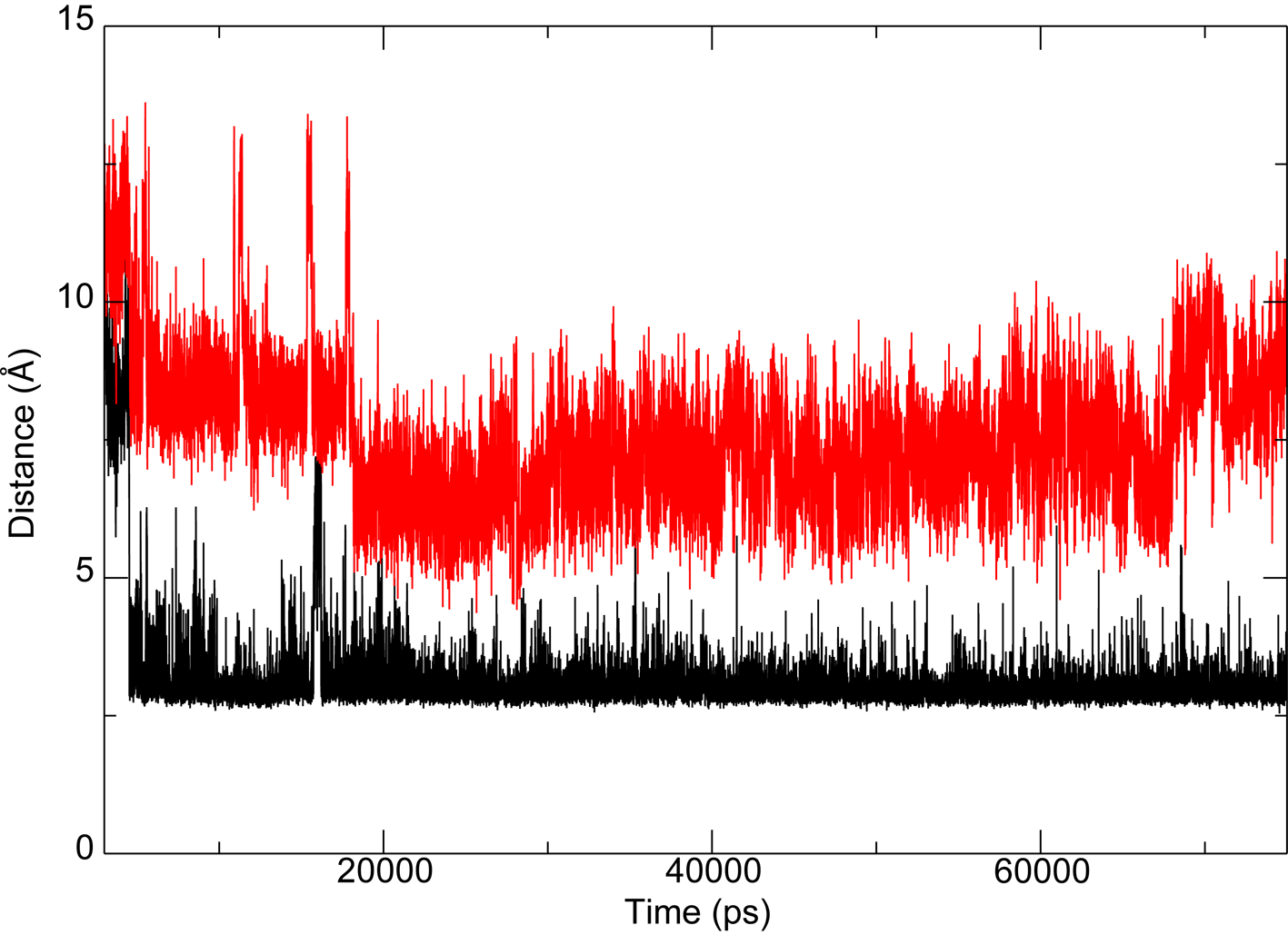

Supplement: Figure S6 — Time evolution of the distance between Lys532 and Gua +1. Atomic distance as a function of time between the Nζ of Lys532 and the O5′ atom of Gua +1. The trajectories of the binary and ternary complexes are reported in black and red, respectively. (TIF) [file pone.0051354.s006.tif]
